# Anti-Melanogenic Potential of Malabar Spinach (*Basella alba*) in Human Melanoma Cells with Oxidative Stress Suppression and Anti-Inflammatory Activities

**DOI:** 10.3390/foods13182943

**Published:** 2024-09-18

**Authors:** Pichchapa Linsaenkart, Warintorn Ruksiriwanich, Korawan Sringarm, Chaiwat Arjin, Pornchai Rachtanapun, Chuda Chittasupho, Juan M. Castagnini, Romchat Chutoprapat, Anja Mueller, Korawinwich Boonpisuttinant

**Affiliations:** 1 Department of Pharmaceutical Sciences, Faculty of Pharmacy, Chiang Mai University, Chiang Mai 50200, Thailand; pichchapa_li@cmu.ac.th (P.L.); chuda.c@cmu.ac.th (C.C.); 2 Cluster of Valorization and Bio-Green Transformation for Translation Research Innovation of Raw Materials and Products, Chiang Mai University, Chiang Mai 50200, Thailand; korawan.s@cmu.ac.th; 3 Cluster of Agro Bio-Circular-Green Industry (Agro BCG), Chiang Mai University, Chiang Mai 50200, Thailand; pornchai.r@cmu.ac.th; 4 Department of Animal and Aquatic Sciences, Faculty of Agriculture, Chiang Mai University, Chiang Mai 50200, Thailand; chaiwat.arjin@cmu.ac.th; 5 School of Agro-Industry, Faculty of Agro-Industry, Chiang Mai University, Chiang Mai 50100, Thailand; 6 Research Group in Innovative Technologies for Sustainable Food (ALISOST), Department of Preventive Medicine and Public Health, Food Science, Toxicology and Forensic Medicine, Faculty of Pharmacy, Universitat de València, Avenida Vicent Andrés Estellés s/n, 46100 Burjassot, Spain; juan.castagnini@uv.es; 7 Department of Pharmaceutics and Industrial Pharmacy, Faculty of Pharmaceutical Sciences, Chulalongkorn University, Bangkok 10300, Thailand; romchat.c@pharm.chula.ac.th; 8 School of Pharmacy, University of East Anglia, Norwich NR4 7TJ, UK; anja.mueller@uea.ac.uk; 9 Innovative Natural Products from Thai Wisdoms (INPTW), Faculty of Integrative Medicine, Rajamangala University of Technology Thanyaburi, Pathumthani 12130, Thailand; korawinwich_b@rmutt.ac.th

**Keywords:** antioxidant, anti-inflammation, anti-melanogenesis, *Basella alba*, caffeic acid, catechin, *p*-coumaric acid

## Abstract

*Basella alba* has been used in Thai remedies to treat skin disorders, but scientific evidence supporting its efficacy is currently limited. In this study, we investigated the inhibitory effects of *B. alba* extracts on melanin production using melanoma cells, as well as their impact on oxidative stress and inflammation in keratinocytes. The results demonstrate that *B. alba* extracts inhibited melanin content and cellular tyrosinase activity in 3-isobutyl-1-methylxanthine (IBMX)-induced melanoma cells by downregulating *MITF* and the pigmentary genes *TYR*, *TRP-1*, and *DCT.* Interestingly, the *MITF* regulator gene was inhibited by both the 50% and 95% ethanolic extracts of *B. alba* with levels of 0.97 ± 0.19 and 0.92 ± 0.09 of the control, respectively, which are comparable to those observed in the arbutin treatment group at 0.84 ± 0.05 of the control. Moreover, after hydrogen peroxide (H_2_O_2_) exposure, pretreatment with *B. alba* reduced lipid peroxidation byproducts and increased the levels of antioxidant-related genes, including *SOD-1*, *GPX-1*, and *NRF2*. Notably, the suppression of the *POMC* promoter gene in keratinocytes was observed, which may disrupt melanogenesis in melanocytes involving the *MC1R* signaling pathway. *MC1R* mRNA expression decreased in the treatments with 50% and 95% ethanolic extracts of *B. alba*, with relative levels of 0.97 ± 0.18 and 0.90 ± 0.10 of the control, respectively, similar to the arbutin-treated group (0.88 ± 0.25 of control). A significant reduction in nitric oxide was also observed in the *B. alba*-treated groups, along with a decrease in genes associated with pro-inflammatory cytokines, including *IL-1β*, *IL-6*, and *COX-2*. These findings suggest that *B. alba* has potential in the prevention of skin-related problems.

## 1. Introduction

*Basella* spp. (Malabar spinach) is one of the climbing vine plants from the *Basellaceae* family, which is widely distributed in Asia and Africa. There are two species, namely *Basella alba* (green stem) and *Basella rubra* (red stem), which normally grow in the northern and northeastern parts of Thailand and are used as ingredients in soup, stir-fry dishes, and side dishes. In both traditional Thai and Ayurvedic remedies, the aerial parts of Malabar spinach are employed in the treatment of dermatological conditions, including urticaria, skin rashes, burns, and ringworm. Furthermore, they are utilized for the prevention of acne, freckles, and potentially melanoma [1]. The primary compounds identified in *B. alba* are phenolics, flavonoids, and terpenoids [2,3]. Along with scientific evidence, *B. alba* has been shown to possess antioxidant properties, reduce cholesterol levels [4], and exhibit antiproliferative effects against colorectal cancer [5]. In addition, it is utilized as a biomaterial in wound care [6]. 

The epidermis at the skin’s surface functions as the primary defense, protecting the skin barrier against environmental stimuli. The upper layer of the epidermis is composed of keratinocytes, with each melanocyte surrounded by approximately 36 keratinocytes [7]. External stressors, including radiation, airborne particles, and influences from the skin microbiome, affect melanocytes and keratinocytes, leading to increased oxidative stress and the release of pro-inflammatory cytokines. Persistent exposure to environmental factors interferes with the cellular interactions between skin cells. This disruption leads to an imbalance in the antioxidant system, along with an increase in various pro-inflammatory cytokines, which in turn contributes to skin cell senescence. Consequently, the aging process of the skin is distinctly characterized by changes in pigmentation, the formation of wrinkles, reduced skin elasticity, and delayed wound healing [8,9,10]. Keratinocytes are the principal cells responsible for both signaling and regulating the process of melanogenesis. In response to UV radiation, air particulate matter, or inflammatory chemokines, the pro-opiomelanocortin (*POMC*) gene promoter in keratinocytes is activated, resulting in the production of several peptides, such as adrenocorticotropic hormone (ACTH) and α-melanocyte-stimulating hormone (α-MSH). These peptides bind to the melanocortin-1 receptor (MC1R) on adjacent melanocytes, thereby leading to increased melanin production through the cyclic adenosine monophosphate (cAMP) signaling pathway [11,12]. Elevated cAMP levels serve as a second messenger to activate protein kinase A (PKA). PKA subsequently translocate to the nucleus and phosphorylate cAMP response element-binding protein (CREB), thereby activating the melanocyte-inducing transcription factor (*MITF*). The *MITF* transcription factor then regulates the pigmentation-related genes, including tyrosinase (*TYR*), tyrosinase-related protein-1 (*TRP-1*), and tyrosinase-related protein-2, also referred to dopachrome tautomerase (*TRP-2* or *DCT*) [7]. Melanin is synthesized from the substrates, L-tyrosine and L-dihydroxyphenylalanine (L-DOPA). This process involves a series of enzymatic reactions mediated by TYR, TRP-1, and DCT. Typically, melanin plays a crucial role as a UV absorber, antioxidant, and radical scavenger [7]. However, chronic exposure to external stimuli without protection can lead to skin hyperpigmentation disorders, such as melasma, freckles, or solar lentigines. Persistent hyperpigmentation and DNA damage may potentially progress to cutaneous carcinoma [13]. The roles of detoxification enzymes, such as superoxide dismutase (SOD), catalase (CAT), glutathione peroxidase (GPX), reduced glutathione (GSH), or heme oxygenase-1 (HO-1), are regulated by a major transcription factor, nuclear factor erythroid 2-related factor 2 (NRF2) in protecting against oxidative stress. A previous skin biopsy study has demonstrated that melasma skin lesions have decreased levels of NRF2, which is related to the presence of oxidative stress [14]. In addition to the downregulation of NRF2 signaling, the levels of pro-inflammatory cytokines, including tumor necrosis factor alpha (TNF-α), cyclooxygenase-2 (COX-2), interleukin (IL)-6, IL-1β, and IL-8, are notably elevated in keratinocyte cells [15]. 

To date, there are limitations associated with the anti-melanogenic efficacy of single therapeutic agents, such as N-acetylcysteine, or vitamin E alone, which have proven insufficient for the treatment of melasma. The combination of antioxidant compounds, such as vitamin E and ascorbic acid, with crude extracts derived from herbs appears to synergistically enhance the treatment of skin cell aging and hyperpigmentation [9]. 

Therefore, future research on plant extracts is expected to reveal significant potential for addressing dermatological disorders through their synergistic effects. Currently, there is no scientific evidence supporting the biological effects of *B. alba* extract on melanogenesis. This study aimed to identify the phytochemical composition in *B. alba* crude extracts and evaluate their biological activities, especially regarding melanin levels and tyrosinase activity in a human melanoma model. Additionally, we have assessed the regulation of genes associated with melanogenesis, including the transcription factor gene *MITF*, pigmentary genes, such as *TYR*, *TRP-1*, and *DCT*, and the receptor for bioactive peptides released from keratinocytes, *MC1R*. Furthermore, we have determined the effects of *B. alba* on antioxidant and anti-inflammatory responses in human keratinocyte cells, focusing on gene expression related to the antioxidant system (*SOD-1*, *GPX-1*, and *NRF2*), pro-inflammatory cytokines (*IL-1β*, *IL-6*, and *COX-2*), and the *POMC* promoter gene, which regulates the paracrine effects between keratinocytes and melanocytes. 

## 2. Materials and Methods 

### 2.1. Collection and Extraction of B. alba Leaves

The leaves of *B. alba* were collected from a local farm in Lamphun Province, Thailand in June 2023 and identified by the Pharmaceutical and Natural Products Research and Development Unit (PNPRDU), Chiang Mai University, Chiang Mai, Thailand. Herbarium no. PNPRDU65011 was kept in the PNPRDU. The samples were cleaned, air-dried, crushed into powder, and stored at 4 °C before extraction. The plant materials (2000 g) were macerated with different solvents, including distilled water, 50% ethanol, and 95% ethanol (1:2 *w/v*) at room temperature for 48 h. The extraction was repeated twice. The extract solution was then filtered using Whatman filter paper no. 1. The extraction solvent was removed by a freeze-dryer or rotary evaporator. The resulting aqueous extract was subjected to a freeze-dryer (Beta 2–8 LD-plus, Martin Christ Gefriertrocknungsanlagen GmbH, Osterode am Harz, Germany), while the resulting 50% ethanol and 95% ethanol extracts were evaporated at 50 °C by an evaporator (Hei-VAP value, Heidolph, Schwabach, Germany) until the solvent was completely removed. The aqueous, 50% ethanolic, and 95% ethanolic extracts of *B. alba* were accordingly labeled as Water, 50 EtOH, and 95 EtOH.

### 2.2. Phenolic Profiles of Crude Extracts by Liquid Chromatography (LC) Coupled with Electrospray Ionization Mass Spectrometry (ESI/MS)

The polyphenol profiles were determined following previous studies [16,17], with slight modifications. LC-ESI/MS analysis was conducted using an Agilent 1260 Infinity II series, coupled with an ESI quadrupole MS 6130 detector (Agilent Technologies, Santa Clara, CA, USA). Separation was carried out on a Restek Ultra C18 reverse-phase column (250 × 4.6 mm, 5 µm, Restek, Bellefonte, PA, USA). The system was operated in gradient mode at 40 °C with a flow rate of 0.5 mL/min. The mobile phase consisted of 0.2% acetic acid in 95% water with 5% methanol (Solvent A) and 0.2% acetic acid in 50% water with 50% acetonitrile (Solvent B). A linear gradient was applied as follows: 0–45 min, 10–20% B; 45–85 min, 20–55% B; 85–97 min, 55–100% B; 97–110 min, 100% B. The injection volume was 20 μL. The spectra were acquired in the negative selected ion monitoring (SIM) mode. The capillary and nozzle voltages were set to −3.5 V. Nitrogen nebulizer gas was used at a flow rate of 12 L/min, with the dissolving line set to 250 °C and the block source set to 400 °C. A fragmentor voltage of 70 V was applied, and a full mass scan was performed from *m*/*z* 100 to 1200 with a scan speed of 250 ms/spectrum. The spectra were analyzed using OpenLab CDS software Rev.C.01.07 SR3 (465) (Agilent Tech., Santa Clara, CA, USA).

### 2.3. Preparation of Stock Solutions of Extracts for Bioassay

All tested samples were dissolved to prepare a stock solution with a concentration of 10 mg/mL and then filtered using 0.02 μm pore-sized syringe filters. The sterile extract solution was then diluted with culture medium, either MEM or DMEM medium, to achieve a concentration range of 0.0078–1 mg/mL. 

### 2.4. Cell Culture and Cytotoxic Assay

G-361 human melanoma cells (IFO50009, JCRB cell bank, Osaka, Japan) were grown in Eagle’s minimal essential medium (Gibco, Grand Island, NY, USA) supplemented with 10% fetal bovine serum (HyClone™ Cytiva, Pasching, Austria) at 37 °C with 5% CO_2_. Human keratinocyte HaCaT cells, a kind gift from Assoc. Prof. Dr. Chuda Chittasupho (Faculty of Pharmacy, Chiang Mai University), were cultured in Dulbecco’s modified Eagle’s medium (Gibco, Grand Island, NY, USA) supplemented with 10% fetal bovine serum and maintained in a humidified incubator 37 °C with 5% CO_2_. The cytotoxicity of the extracts was determined using sulforhodamine B (SRB, Sigma Chemical, St. Louis, MO, USA) dye staining [18]. In brief, the prepared dilutions were incubated with the cells for 48 h. After incubation, the cells were washed with PBS and fixed with 50% trichloroacetic acid (PanReac AppliChem, Barcelona, Spain) at 4 °C for 1 h. The plates were left to dry overnight. Then, the cells were stained by SRB (0.04% *w/v*) for 30 min. The dye was dissolved by 10 mM tris base solution (Vivantis, Selangor, Malaysia) to measure the absorbance at 515 nm. The percentage of cell viability was determined relative to untreated cells.

### 2.5. Melanogenesis Assays 

#### 2.5.1. Melanin Content in 3-Isobutyl-1-Methylxanthine (IBMX)-Induced Melanoma Cells

A melanin content assay was performed based on a previous method [19]. G-361 melanoma cells (2.5 × 10^5^ cells/well) were cultivated in 6-well plates. When confluent, the medium was removed and replaced with *B. alba* extract solution. After 1 h of incubation, 50 μM of 3-isobutyl-1-methylxanthine (IBMX, PanReac AppliChem, Barcelona, Spain) was added to induce melanin production, and the incubation was continued for 48 h. Subsequently, melanin within the cells was dissolved in 1 N NaOH with 10% DMSO at 80 °C for 30 min and measured at 405 nm.

#### 2.5.2. Assessment of Intracellular Tyrosinase Activity 

The intracellular tyrosinase activity assay was performed as previously described [19]. The cells were pretreated with *B. alba* extract solution and then stimulated with 50 µM IBMX for 48 h. The cells were lysate using 1% Triton X-100 (VWR Life Science, Solon, OH, USA) and frozen for 30 min. Subsequently, the cell suspension was centrifuged to obtain the cell supernatant. The obtained supernatant was reacted with the substrate L-DOPA (Sigma Chemical, St. Louis, MO, USA) at 5 mM in phosphate buffer and incubated at 37 °C for 1 h. The formation of L-dopachrome was detected at 475 nm. 

### 2.6. Thiobarbituric Acid Reactive Substance (TBARS) Quantification in Hydrogen Peroxide (H_2_O_2_)-Induced Keratinocyte Cells

The levels of TBARS, one of the reactive substances of the lipid peroxidation process, were determined as previously described [20]. Briefly, HaCaT cells were pre-exposed to *B. alba* extracts or L-ascorbic acid (L-AA), and then incubated with 200 μM H_2_O_2_. The cells were then collected and mixed with a hot thiobarbituric acid (TBA) solution (BDH Chem. Ltd., Poole, UK). The mixture was moved to the freezer to stop the reaction and measured at 532 nm. 

### 2.7. Nitric Oxide (NO) Quantification in Lipopolysaccharide (LPS)-Induced Keratinocyte Cells

The levels of nitric oxide (NO) production from keratinocyte cells after stimulation with lipopolysaccharides (LPS) were indirectly quantified by estimating the levels of nitrite released from the cells, as previously described [20]. Briefly, HaCaT cells at a density of 1 × 10^3^ cells/well were plated in 96-well plates. The cells were pretreated with *B. alba* extracts or diclofenac sodium (DFN) for 1 h and then induced with LPS at 1 μg/mL (Sigma Chemical, St. Louis, MO, USA) for a further 24 h period. The cell supernatants were collected, and their NO levels were determined (Griess Reagent Kit, Invitrogen, Thermo Fisher Scientific, Eugene, OR, USA, cat no. G7921). The levels of nitrite accumulation were calculated using the standard curve equation for sodium nitrite, within a range of 1–100 μM (y = 0.0113x, R^2^ = 0.999).

### 2.8. Gene Expression Analysis by Semi-Quantitative Reverse Transcription–Polymerase Chain Reaction (PCR)

Gene expression analysis was performed as previously described [20]. The total RNA was harvested from the cells using the E.Z.N.A.^®^ and extracted using the Total RNA Kit I (Omega Bio-Tek, Norcross, GA, USA). The NanoDrop^TM^ One^C^ Microvolume UV-Vis Spectrophotometer (Thermo Fisher Scientific, Waltham, MA, USA) was used to quantify the concentration of RNA. For cDNA synthesis, reverse transcription was performed using the MyTaq^TM^ One-Step RT-PCR Kit (Meridian Bioscience^TM^, BIO-65049, Memphis, TN, USA). The nucleic acid was amplified using the DW-T960 Gradient PCR Thermal Cycler (Drawell, Shanghai, China). The expression levels of the targeted genes were normalized to *GAPDH*. Subsequently, the RT-PCR products were separated using agarose gel electrophoresis. Gene expression measurement was carried out using the Gel Doc^TM^ EZ System, and the calculations were performed using Image Lab^TM^ software version 6.1. Table 1 illustrates the list of genes related to melanogenesis, antioxidant, and inflammation.

### 2.9. Statistical Analysis

All quantitative results are presented as the mean ± standard deviation (SD). The data were subjected to one-way ANOVA to assess the overall differences among the groups, followed by the LSD post hoc test to identify specific pairwise comparisons. Statistical significance was conducted using SPSS 23.0 software (Chicago, IL, USA), with values of *p* < 0.05 considered statistically significant. 

## 3. Results 

### 3.1. Crude Extract Preparation and Phytochemical Compositions

The yields of the crude extract preparations from the three different solvents were 6.20%, 13.05%, and 8.50% *w/w* based on dry materials for the aqueous, 50% ethanolic, and 95% ethanolic extracts of *B. alba*, respectively. As shown in Table 2, the phenolic profiles of the crude extracts were quantified using LC-ESI/MS. Catechin was detected in high proportions in the aqueous and 50% ethanolic extracts. On the contrary, the caffeic acid content from the ethanolic extraction was higher than that from the aqueous extraction. Interestingly, *p*-coumaric acid was found to be the most abundant in the 95% ethanolic extract. According to previously published data, the leaves of *B. alba* contain higher levels of total phenolic and flavonoid compounds compared to *B. rubra*, resulting in superior chelating ability against iron ions [2]. It has been reported that methanol extraction can extract a broader range of active compounds from *B. alba*, such as 10-(methoxycarbonyl)-N-acetylcolchinol, known for its anti-cancer properties, and propanoic acid derivatives, recognized for their anti-diabetic effects [5]. However, the use of methanol for topical application is not considered safe.

### 3.2. Effects of Basella alba Extracts on Cell Viability

**Figure 1:**
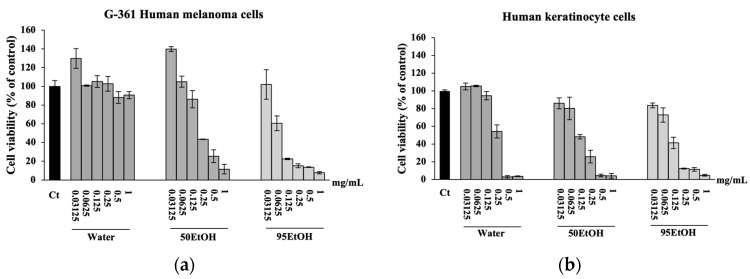
Effects of *Basella alba* extracts on cell viability of (**a**) G-361 human melanoma and (**b**) human keratinocyte HaCaT cells. Data are presented as mean ± SD (*n* = 3). Water: aqueous extract of *Basella alba*; 50 EtOH: 50% ethanolic extract of *Basella alba*; 95 EtOH: 95% ethanolic extract of *Basella alba*.

We initially assessed the viability of G-361 human melanoma and human keratinocyte HaCaT cells exposed to *B. alba* extracts (Figure 1). The results of the SRB assay indicate that treatment with *B. alba* extracts reduced human melanoma and keratinocyte cells in a dose-dependent manner. The maximum concentration of each extract that resulted in cell viability of more than 80% was selected to further tests [20]. The results demonstrate that the percentages of viable G-361 cells following treatment with Water, 50 EtOH, and 95 EtOH at 0.03125 mg/mL were 129.83 ± 10.42%, 139.77 ± 2.61%, and 102.05 ± 15.66% of the control, respectively. Furthermore, all *B. alba* extracts at 0.0625 mg/mL caused approximately a 30% to 40% decrease in G-361 cell viability (Figure 1a). Similarly, the effects of Water, 50 EtOH, and 95 EtOH at 0.03125 mg/mL on HaCaT cell viability were 104.90 ± 3.83%, 85.96 ± 6.08%, and 83.63 ± 2.66% of the control, respectively. Additionally, all *B. alba* extracts at 0.0625 mg/mL slightly decreased HaCaT cell viability, with percentages of 105.57 ± 0.83%, 80.19 ± 12.80%, and 72.79 ± 8.04% of the control, respectively (Figure 1b). These findings suggest that the *B. alba* extract concentrations of 0.03125 mg/mL or lower were non-cytotoxic to both cell types. Therefore, this concentration was chosen for further experiments in our study.

### 3.3. Basella alba Extracts Suppress Melanin Content, Tyrosinase Activity, and Expression of Melanogenesis-Related Genes in IBMX-Treated Melanoma Cells

**Figure 2:**
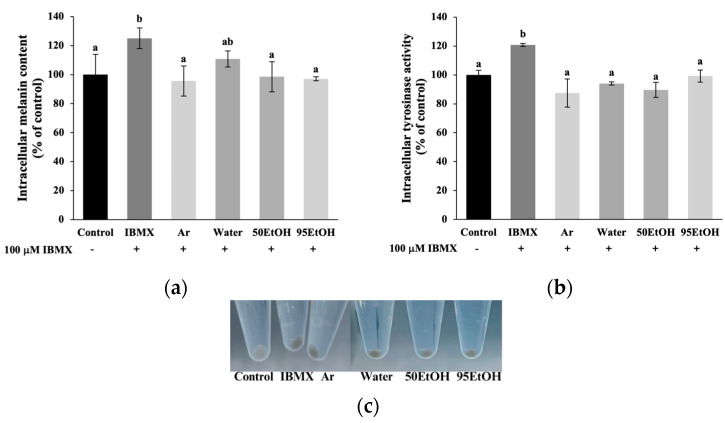
Effects of *Basella alba* extracts on (**a**) melanin production, (**b**) cellular tyrosinase activity in IBMX-induced human melanoma cells and (**c**) appearance of cell pellets after exposure to different samples. Data are presented as mean ± SD (*n* = 3). Statistical significance in the comparison among all extracts is indicated by different letters (a and b), with significance defined as *p* < 0.05, as established through one-way ANOVA and subsequent LSD post hoc comparison. IBMX: 3-isobutyl-1-methylxanthine; Ar: arbutin; Water: aqueous extract of *Basella alba*; 50 EtOH: 50% ethanolic extract of *Basella alba*; 95 EtOH: 95% ethanolic extract of *Basella alba*.

Melanin production and intracellular tyrosinase activity assays were performed by pre-exposing melanoma cells to *B. alba* extracts, followed by induction with IBMX for an additional 48 h. The levels of intracellular melanin and tyrosinase activity were significantly higher in IBMX-treated melanoma cells than in the control group, with values of 125.00 ± 7.09% and 120.74 ± 1.05% of the control, respectively (*p* < 0.05). The treatments with the 50% and 95% ethanolic *B. alba* extracts resulted in significant decreases in melanin production in the IBMX-induced melanoma cells to 98.53 ± 10.40% and 97.06 ± 1.39% of the control, respectively (*p* < 0.05) (Figure 2a). Additionally, the aqueous, 50%, and 95% ethanolic extracts of *B. alba* demonstrated significant inhibitory effects on cellular tyrosinase activity in the IBMX-induced melanoma cells, with values of 94.07 ± 1.05%, 89.63 ± 5.24%, and 99.26 ± 4.19% of the control, respectively (*p* < 0.05) (Figure 2b). Our data indicate that the *B. alba* treatment exhibited anti-melanogenic properties at a comparable level to the standard arbutin.

**Figure 3:**
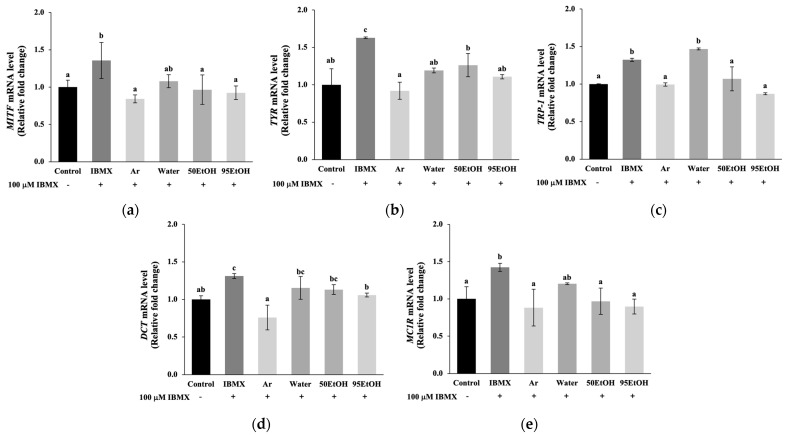
Effects of *Basella alba* extracts on mRNA expression of (**a**) *MITF*, (**b**) *TYR*, (**c**) *TRP-1*, (**d**) *DCT*, and (**e**) *MC1R* in IBMX-induced human melanoma cells. Data are presented as mean ± SD (*n* = 3). Statistical significance in the comparison among all extracts is indicated by different letters (a, b, and c), with significance defined as *p* < 0.05, as established through one-way ANOVA and subsequent LSD post hoc comparison. IBMX: 3-isobutyl-1-methylxanthine; Ar: arbutin; Water: aqueous extract of *Basella alba*; 50 EtOH: 50% ethanolic extract of *Basella alba*; 95 EtOH: 95% ethanolic extract of *Basella alba*.

The effect on gene expression associated with the melanogenesis pathway was further evaluated, including the main transcription factor *MITF*, pigmentary genes *TYR*, *TRP-1*, and *DCT*, and a driver of melanogenesis, *MC1R*. The exposure of melanoma cells to IBMX resulted in a marked upregulation of genes related to melanogenesis compared to untreated cells (*p* < 0.05). Obviously, the 50% and 95% ethanolic extracts of *B. alba* significantly reduced the expression levels of the regulator gene *MITF*, with relative expression levels of 0.97 ± 0.19 and 0.92 ± 0.09, respectively, which are comparable to the reduction observed with arbutin at 0.84 ± 0.05 (Figure 3a). In the IBMX-induced melanoma cells, pretreatment with the aqueous, 50%, and 95% ethanolic extracts of *B. alba* significantly inhibited the expression of tyrosinase, encoded by the *TYR* gene, to 1.19 ± 0.03, 1.26 ± 0.15, and 1.11 ± 0.03, respectively, which are comparable to the levels observed in the arbutin treatment group (0.92 ± 0.12) (Figure 3b). Notably, the 50% and 95% ethanolic extracts of *B. alba* significantly reduced the expression levels of the pigmentary gene *TRP-1* to 1.07 ± 0.16 and 0.87 ± 0.01, respectively, in the IBMX-stimulated melanoma cells (*p* < 0.05). These effects were comparable to those obtained from the standard arbutin treatment, which resulted in *TRP-1* expression levels of 0.99 ± 0.02 (Figure 3c). As demonstrated in Figure 3d, the expression of the pigmentary gene *DCT* was decreased in the groups treated with 95% ethanolic extract of *B. alba* and arbutin, with relative expression levels of 1.06 ± 0.03 and 0.76 ± 0.17, respectively. In addition, the expression of the *MC1R* gene, which is associated with paracrine effects from keratinocyte signaling, was significantly lower in the groups treated with 50% and 95% ethanolic extracts of *B. alba*, with relative expression levels of 0.97 ± 0.18 and 0.90 ± 0.10, respectively, compared to the IBMX- stimulated melanoma cells, which had a relative expression level of 1.42 ± 0.06 (*p* < 0.05). These effects were comparable to those obtained from the standard arbutin treatment (0.88 ± 0.25), as shown in Figure 3e. Taken together, these data suggest that 50 EtOH and 95 EtOH can alleviate melanogenesis in human melanoma cells with inhibitory effects against *MITF*, *TYR*, *TRP-1*, and *MC1R* mRNA expression.

### 3.4. Basella alba Extracts Alleviate TBARS Formation by Stimulating Expression of Genes Related to Antioxidant Pathways in H_2_O_2_-Treated Keratinocytes

**Figure 4:**
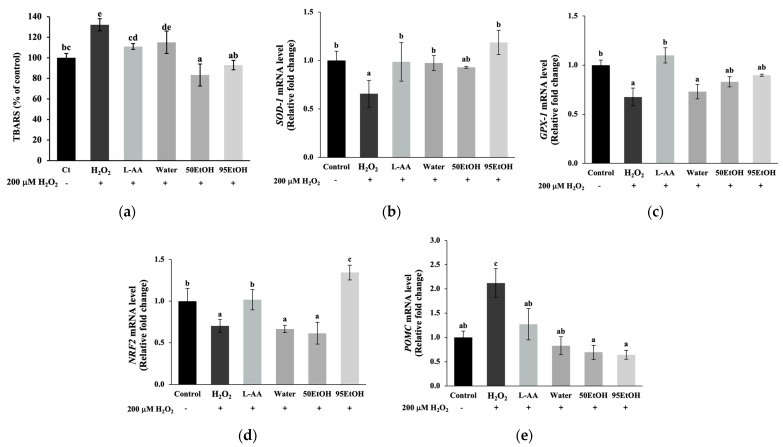
Effects of *Basella alba* extracts on (**a**) TBARS production and mRNA expression of (**b**) *SOD-1*, (**c**) *GPX-1*, (**d**) *NRF2*, and (**e**) *POMC* in H_2_O_2_-induced human keratinocyte cells. Data are presented as mean ± SD (*n* = 3). Statistical significance in the comparison among all extracts is indicated by different letters (a, b, c, d, and e), with significance defined as *p* < 0.05, as established through one-way ANOVA and subsequent LSD post hoc comparison. H_2_O_2_: hydrogen peroxide; L-AA: L-ascorbic acid; Water: aqueous extract of *Basella alba*; 50 EtOH: 50% ethanolic extract of *Basella alba*; 95 EtOH: 95% ethanolic extract of *Basella alba*.

In H_2_O_2_-induced keratinocytes, the level of the lipid peroxidation product TBARS significantly increased to 132.18 ± 5.91% of the control compared to untreated cells (*p* < 0.05). Treatment with the 50% and 95% ethanolic extracts of *B. alba* resulted in a significant decrease in TBARS formation to 83.33 ± 10.71 and 92.93 ± 4.63% of the control, respectively (*p* < 0.05) (Figure 4a). Together with the mRNA expression of genes associated with antioxidant defenses, exposure to H_2_O_2_ suppressed the levels of *SOD-1*, *GPX-1*, and *NRF2*, resulting in relative fold changes of 0.66 ± 0.14, 0.68 ± 0.09, and 0.70 ± 0.07, respectively (*p* < 0.05). We found that the treatment with all extracts increased the relative expression of *SOD-1* to 0.98 ± 0.07, 0.93 ± 0.01, and 1.18 ± 0.12, respectively (Figure 4b). However, the *B. alba* extracts did not influence *GPX-1* expression, as shown in Figure 4c. A notably significant increase in transcription factor *NRF2* was observed with the 95% ethanolic extract of *B. alba*, reaching a level of mRNA expression of 1.34 ± 0.09 in the H_2_O_2_-induced keratinocytes (*p* < 0.05) (Figure 4d).

To evaluate the paracrine interactions between keratinocyte and melanocyte cells, the promoter gene *POMC* was investigated. As illustrated in Figure 4e, cellular oxidation after exposure to H_2_O_2_ contributed to a notable increase in the mRNA expression of *POMC* to 2.12 ± 0.29 compared to the control group (*p* < 0.05). A significant reduction in *POMC* expression was observed in all *B. alba* treatment groups, with mRNA relative expression levels of 0.83 ± 0.18, 0.69 ± 0.15, and 0.64 ± 0.09, respectively (*p* < 0.05).

### 3.5. Basella alba Extracts Inhibit NO Production and Downregulate Expression of Genes Associated with Inflammatory Cytokines in LPS-Treated Keratinocytes

**Figure 5:**
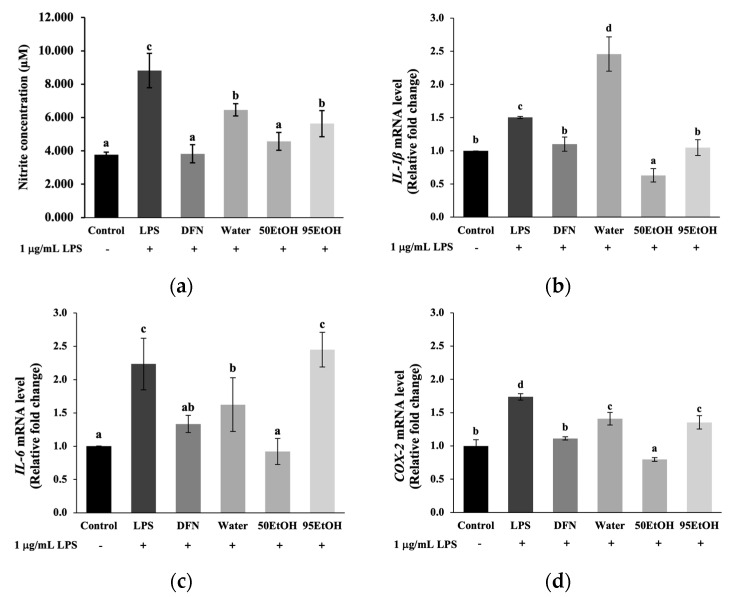
Effects of *Basella alba* extracts on (**a**) nitric oxide production and mRNA expression of (**b**) *IL-1β*, (**c**) *IL-6*, and (**d**) *COX-2* in LPS-induced human keratinocyte cells. Data are presented as mean ± SD (*n* = 3). Statistical significance in the comparison among all extracts is indicated by different letters (a, b, c, and d), with significance defined as *p* < 0.05, as established through one-way ANOVA and subsequent LSD post hoc comparison. LPS: lipopolysaccharide; DFN: diclofenac sodium; Water: aqueous extract of *Basella alba*; 50 EtOH: 50% ethanolic extract of *Basella alba*; 95 EtOH: 95% ethanolic extract of *Basella alba*.

In the present study, we estimated NO production from the levels of nitrite concentrations, as demonstrated in Figure 5a. The tested extracts significantly reduced the nitrite levels to 6.46 ± 0.37, 4.56 ± 0.54, and 5.63 ± 0.79 μM, respectively, compared to the LPS treatment group (8.82 ± 1.03 μM) (*p* < 0.05). Notably, treatment with the 50% ethanolic extract of *B. alba* showed a significantly remarkable decrease in the relative levels of pro-inflammatory mediators, *IL-1β*, *IL-6*, and *COX-2*, to 0.63 ± 0.10, 0.92 ± 0.19, and 0.80 ± 0.03, respectively, in LPS-induced keratinocytes (*p* < 0.05). However, 95 EtOH influenced only the reduction of *IL-1β* (1.05 ± 0.12) to a level comparable to the DFN treatment (1.10 ± 0.12) (Figure 5b). In contrast, the aqueous extract of *B. alba* did not affect the suppression of inflammatory cytokines.

## 4. Discussion 

Hyperpigmentation disorders, notable indicators of chronological skin changes, are linked to the accumulation of senescent skin cells. Melasma, in particular, is a chronic and relapsing dermatological problem generally related to genetic factors, prolonged sun exposure, pregnancy, and the administration of oral contraceptives. Additionally, post-inflammatory hyperpigmentation presents as darkened lesions following trauma or infection. These conditions are characterized by oxidative damage, subclinical inflammation, and multiple markers of early skin aging [8,25]. Notably, elevated *POMC* mRNA expression was found in keratinocytes after H_2_O_2_ exposure (Figure 4e). This response involves the activation of the *POMC* promoter gene in keratinocytes, leading to the production of POMC peptides, such as α-MSH and ACTH. These peptides bind to the MC1R on melanocytes, thereby stimulating melanogenesis. Activation of the MC1R receptor increases cAMP synthesis, which contributes to *MITF* expression. As reported in a previous study, treatment with forskolin, an adenylate cyclase activator, or IBMX, a phosphodiesterase inhibitor, activated the cAMP pathway, resulting in increased melanin produced in melanocytes [26]. In agreement with this, our results indicate that IBMX induction influenced both elevated melanin production and increased tyrosinase activity (Figure 2). This effect is suggested by the increased levels of *MC1R*, which subsequently led to the induction of *MITF* expression (Figure 3e).

The regulator gene *MITF* plays a crucial role in the response of increased melanin production after skin damage. The phosphorylation of *MITF* then activates downstream pigmentary genes, including *TYR*, *TRP-1*, and *DCT*. Primarily, *TYR* encodes the tyrosinase enzyme, which constitutes the rate-limiting process in melanin pigment biosynthesis [7]. Consequently, the melanin produced will be transferred to neighboring keratinocytes in response to cutaneous injury. Excessive melanin accumulation contributes to hyperpigmented lesions and, in severe cases, may progressively lead to melanoma [13]. Our study demonstrated that treatment with *B. alba* extracts resulted in the downregulation of melanin production, along with a reduction in cellular tyrosinase activity, in IBMX-induced melanoma models (Figure 2). Interestingly, ethanolic samples of *B. alba* exhibited markedly inhibitory effects on *MITF*, *TYR*, and *TRP-1* (Figure 3a–c).

Exposure to extrinsic factors, such as solar damage or air pollution, results in the premature aging of human skin cells, particularly keratinocytes, through the generation of reactive oxygen species (ROS). The presence of various ROS, such as superoxide anion radical, singlet oxygen, and H_2_O_2_, leads to interactions with biomolecules that impair cell signaling and impact the survival and function of skin cells. The accumulation of H_2_O_2_ triggers the process of lipid peroxidation, targeting the plasma membrane. This process subsequently produces malondialdehyde (MDA), a byproduct of lipid peroxidation [27]. MDA released from the cells can react with TBA to form TBARS, which can be quantified. As illustrated in Figure 4, the TBARS levels were significantly higher in the H_2_O_2_-induced keratinocytes than in the control group. Our findings suggest that stimulation with H_2_O_2_ downregulated the expression of *NRF2* associated with decreased expression levels of antioxidant-related genes, including *SOD-1* and *GPX-1*. *NRF2* has been identified as a crucial regulator of antioxidant responses. A previous three-dimensional skin study established that an *NRF2*-overexpressing skin cell model can restore the activities of SOD, CAT, GPX, and GSH, accompanied by a reduction in ROS and malondialdehyde [28]. In the present study, we observed a significant decline in TBARS levels after pre-treatment with 50 EtOH and 95 EtOH compared to H_2_O_2_-induced keratinocytes (Figure 4a). Additionally, pre-treatment with a 95% ethanolic extract of *B. alba* was able to restore the expression levels of the gene encoding phase 2 antioxidant enzyme *SOD-1* and the regulator gene *NRF2* (Figure 4b,d).

Senescent keratinocytes exhibit a characteristic known as the senescence-associated secretory phenotype (SASP), characterized by increased production of pro-inflammatory cytokines, including TNF-α, IL-1α, IL-1β, IL-6, and COX-2. These cytokines contribute to chronic inflammation and tissue remodeling in the skin [8,29,30]. According to previously published data, these cytokines and inflammatory mediators, such as nitric oxide, induce melanin production, which contributes to the pathogenesis of post-inflammatory hyperpigmentation [25,31]. In addition, the presence of IL-1β can upregulate the expression of TYR and TRP-1 in mouse melanomas [32]. In this study, all *B. alba* extracts were able to suppress nitric oxide production. Notably, treatment with the 50% ethanolic extract of *B. alba* significantly reduced the gene expression levels of *IL-1β*, *IL-6*, and *COX-2*, while the 95% ethanolic extract influenced the suppression of *IL-1β* (Figure 5). The 95% ethanolic extract lowered *IL-1β* levels similarly to the anti-inflammatory agent DFN-treated group (*p >* 0.05). Interestingly, the 50% ethanolic extract exhibited a stronger inhibitory effect on *IL-1β* than the DFN treatment group (*p* < 0.05) (Figure 5b).

There is a strong relationship between polyphenol molecules from the diet and their antioxidant effects, which occur through the bonding of polyphenol hydroxyl groups with radicals [33]. Together with our findings, previous research has also reported that the activation of NRF2 can reduce the paracrine factors released from keratinocytes, leading to the downregulation of key signaling pathways, such as cAMP/CREB/MITF, involved in melanogenesis in melanocytes. Therefore, natural NRF2 modulators could offer a promising strategy for preventing and treating hyperpigmentation disorders [15]. To illustrate, catechin, a natural flavonoid, directly chelates metal ions and scavenges ROS and reactive nitrogen species (RNS), while also indirectly modulating the upregulation of antioxidant enzymes. Moreover, in animal studies, the oral administration of catechin in mice substantially augmented the enzymatic activities of SOD, CAT, and GSH [34]. Consistent with previous research, the pre-exposure of B16F10 mouse melanoma cells to caffeic acid prior to UV radiation influenced the NRF2 levels, resulting in increased expression of NRF2 and targeted antioxidants, such as glutathione S-transferase (GST) and glutamate cysteine ligase catalytic subunit (GCLC), thereby diminishing photo-induced melanin synthesis [35]. In our present study, the phenolic contents were measured by LC-ESI/MS. High levels of catechin were found in the Water and 50 EtOH extracts. The caffeic acid content was greater in the ethanol extraction compared to the aqueous extraction. Notably, *p*-coumaric acid was found at the highest concentration in the 95% ethanolic extract (Table 2). 

A previous study suggested that catechin and its derivatives, along with hydroxycinnamate compounds, act as natural tyrosinase inhibitors [36]. Indeed, catechin derivatives, such as epigallocatechin gallate and gallocatechin gallate, have been proven to reduce tyrosinase activity by downregulating MC1R expression. This occurs through targeting specific residues of the MC1R protein, resulting in decreased levels of MITF and the TYR family [37]. In addition to hydroxycinnamate compounds, caffeic acid and *p*-coumaric acid have also demonstrated inhibitory potential against tyrosinase activity. Computational studies have illustrated that the *o*-diphenolic structure of caffeic acid and n-nonyl caffeate plays a crucial role in interacting with the binding site of the tyrosinase enzyme [38]. Furthermore, the application of *p*-coumaric acid on human skin fragments has shown depigmentation activity through the interaction between the aromatic and acid moieties of *p*-coumaric acid and tyrosinase, as revealed by molecular docking [39]. 

Bioactive compounds present in *B. alba* extracts, including catechin, caffeic acid, and *p*-coumaric acid, exhibit significant anti-melanogenic property. Based on our findings, the 50% ethanolic extract of *B. alba* is the preferred option owing to its superior extraction yield compared to aqueous and 95% ethanolic extraction methods. This extract possessed comparable anti-melanogenic activity and demonstrated a notable capacity to suppress inflammatory cytokines, with its levels of catechin, caffeic acid, and *p*-coumaric acid being within acceptable ranges. Consequently, the phytochemical characterization of *B. alba* should be incorporated into the quality control standards for the extraction process and used as phytochemical markers. However, the findings of this study are primarily based on in vitro cell models, which may not fully replicate the complex physiological environment in vivo, thereby limiting the generalizability of the results. To enhance the relevance of these findings, future research should include animal studies or clinical trials to validate the efficacy and safety of the extracts in more complex biological systems. Additionally, although key phenolic compounds have been identified, the specific roles of these compounds in the observed anti-melanogenic and anti-inflammatory activities require further elucidation. Future studies should focus on isolating, purifying, and characterizing these active components to acquire a more comprehensive understanding of their mechanisms of action. Moreover, this study does not address the long-term effects or potential side effects associated with the use of *B. alba* extracts. Therefore, future research should undertake an evaluation of the long-term safety and efficacy of these extracts, particularly in a clinical setting. Our future research will focus on employing co-culture models and 3D skin equivalents to investigate biomolecules and paracrine effects, aiming to confirm the anti-melanogenic potential of *B. alba* extracts and their bioactive compounds. Overall, the study is well-executed and provides valuable insights into the potential therapeutic applications of *B. alba* for skin-related conditions.

## 5. Conclusions

The leaves of *Basella alba* are rich in phenolic compounds, including catechin, caffeic acid, and *p*-coumaric acid. The anti-melanogenic effects of *B. alba* were mediated through the inhibition of the regulator gene *MITF* and the downregulation of pigmentary genes, such as *TYR*, *TRP-1*, and *DCT*, in the human melanoma cells exposed to cAMP stimulator IBMX. The prevention of epidermal skin damage was also found in the *B. alba* treatment groups, as indicated by a reduction in the lipid peroxidation end products. Additionally, higher expressions of genes related to antioxidant enzymes, including *SOD-1*, *GPX-1*, and *NRF2*, were observed in the groups treated with the *B. alba* extracts in the keratinocytes under H_2_O_2_-induced oxidative stress. The *B. alba* extracts further suppressed the expression of the *POMC* promoter gene in keratinocytes, which regulates paracrine effects associated with melanogenesis in neighboring melanocytes. Notably, these effects were attributed to the anti-melanogenic action mediated through the cellular signaling pathway involving *MC1R*. Moreover, the *B. alba* treatments resulted in decreased accumulation of inflammatory mediators, such as nitric oxide, and a reduction in pro-inflammatory cytokines, including *IL-1β*, *IL-6*, and *COX-2*. Therefore, the effects of *B. alba* extracts exhibited effective anti-melanogenic activity, potent antioxidant potential, and significant suppression of inflammatory cytokines, establishing them as promising candidates for further applications in skin-related studies. 

## Figures and Tables

**Table 1:** Primer sequences used in this study.

Primer	Forward Sequence (5′ to 3′)	Reverse Sequence (5′ to 3′)	Reference
*MITF*	ACCGTCTCTCACTGGATTGGT	ACCAATCCAGTGAGAGACGGT	[20]
*TYR*	TTGGCATAGACTCTTCTTGTTGCGG	CCGCAACAAGAAGAGTCTATGCCAA	[20]
*TRP-1*	TGGCAAAGCGCACAACTCACCC	GGGTGAGTTGTGCGCTTTGCCA	[20]
*DCT*	TGTGGAGACTGCAAGTTTGGC	GCCAAACTTGCAGTCTCCACA	[20]
*MC1R*	GCAGCAGCTGGACAATGTCA	TGACATTGTCCAGCTGCTGC	[21]
*SOD-1*	TGGAGATAATACAGCAGGCT	AGCCTGCTGTATTATCTCCA	[22]
*GPX-1*	AGAAGTGCGAGGTGAACGGT	ACCGTTCACCTCGCACTTCT	[22]
*NRF2*	AAACCAGTGGATCTGCCAAC	GTTGGCAGATCCACTGGTTT	[20]
*POMC*	CCTGCCTGGAAGATGCCGAGAT	ATCTCGGCATCTTCCAGGCAGG	[23]
*IL-1β*	CTGAGCTCGCCAGTGAATG	CATTCACTGGCGAGCTCAG	[20]
*IL-6*	ACTCACCTCTTCAGAACGAATTG	CAATTCGTTCTGAAGAGGTGAGT	[20]
*COX-2*	GGGATTTTGGAACGTTGTGAA	TTCACAACGTTCCAAAATCCC	[24]
*GAPDH*	GGAAGGTGAAGGTCGGAGTC	CTCAGCCTTGACGGTGCCATG	[20]

**Table 2:** Phenolic profiles of *Basella alba* extracts.

Bioactive Compounds (mg/g Extract)	Water	50 EtOH	95 EtOH
Catechin	2.00 ± 0.13 ^b^	1.83 ± 0.03 ^b^	0.92 ± 0.09 ^a^
Caffeic acid	0.08 ± 0.00 ^a^	0.20 ± 0.00 ^b^	0.22 ± 0.00 ^c^
*p*-Coumaric acid	0.51 ± 0.04 ^b^	0.27 ± 0.00 ^a^	0.83 ± 0.00 ^c^

Data are presented as mean ± SD (*n* = 3). Statistical significance in the comparison among all extracts is indicated by different letters (a, b, and c), with significance defined as *p* < 0.05, as established through one-way ANOVA and subsequent LSD post hoc comparison. Water: aqueous extract of *Basella alba*; 50 EtOH: 50% ethanolic extract of *Basella alba*; 95 EtOH: 95% ethanolic extract of *Basella alba*; ND: not detected.

## Data Availability

The original contributions presented in the study are included in the article; further inquiries can be directed to the corresponding author.
